# Can we predict intensive care relatives at risk for posttraumatic stress disorder?

**DOI:** 10.4103/0972-5229.68221

**Published:** 2010

**Authors:** Lalitha Pillai, Supriya Aigalikar, Sunil M. Vishwasrao, S. M. K. Husainy

**Affiliations:** **From:** CARE Hospital, Pune, India

**Keywords:** Hospital anxiety and depression scale score, ICU patient, IES-R score, posttraumatic disorder

## Abstract

**Aims:**

To identify the relatives of the intensive care unit (ICU) patients at risk for developing symptoms of posttraumatic stress disorders.

**Setting:**

A multidisciplinary hospital ICU.

**Design:**

Prospective single center observational study.

**Material and Methods:**

Relatives of patients admitted in the ICU (May06-Nov06) who consented to answer the questionnaire participated in the study. Anxiety was assessed by using the Hospital Anxiety and Depression Scale (HAD) and vulnerability to posttraumatic disorder (PTSD) by using the Impact of Event Scale Revised (IES-R) which was administered on the fifth day of admission and at two months following discharge or death.

**Results:**

During admission, 48% of the relatives had a HAD score >11 and 72% showed IES-R score >26. There was no association of HAD with gender, patient outcome, working status, age of the patient, or mode of payment of the bills. There was significant association of IES-R >26 with trauma admission, HAD score >11 and mode of payment with the relatives of insured being more stressed as compared to those who settled their bills personally. A total of 35% relatives showed symptoms of posttraumatic stress reaction consistent with a high risk of PTSD after two months. Death in the hospital resulted in elevated HAD and IES-R score during admission and at the two month follow-up. Persistence of stress symptoms was more in school drop outs, working relatives, parents and those with initial anxiety score >11.

**Conclusions:**

HAD score greater than 11 was the only factor at admission which could statistically predict a higher PTSD score on follow-up. Adequate counseling of this group of relatives may prevent lasting psychological sequelae of an ICU admission in the relatives of critically ill.

## Introduction

Intensive care admission can be a stressful event for the families of the critically ill patients. With increasing acceptance of the concept of family-centered care, many recent studies have focused on the relatives of ICU patients.[1–11] Pouchard’s prospective multi-centered study of 78 ICUs in France reported more than two-thirds of family members visiting ICU patients as having symptoms of anxiety or depression during the first few days of the hospitalization irrespective of the patient’s clinical outcome i.e., discharge or death.[7] As acute stress of ICU admission can leads to lasting psychological damage, the contributory factors need to be identified so that remedial measures can be undertaken. We decided to study the factors during admission, which could predict the risk for developing PTSD-related symptoms in relatives (next of kin) of ICU patients.

## Materials and Methods

Relatives of patients admitted in the ICU (May–Nov 06) and who consented to answer the questionnaire participated in the study. Anxiety was assessed using the hospitalanxiety and depression (HAD) scale and vulnerability to posttraumatic stress disorder (PTSD) using the Impact of Event Scale Revised (IES-R). They completed the HADS and IES-R after the patient had been in the ICU for five days and then were followed-up with IES-R telephonically at two months post-ICU discharge or death.

### Statistical analysis

All values are reported as mean and SD for normally distributed variables. Data is presented in actual numbers and percentages. If a difference was found, a paired *t*-test was used to compare groups at each time point. *P* value <0.05 was considered significant and <0.01 considered as highly significant. Data were analyzed using *SPSS 12.0* software.

### Procedure

The Impact of Event Scale Revised (IES-R) is a standardized measure of PTSD symptoms.[11] It is a 22-item scale, which taps into intrusion, avoidance and hyperarrousal symptoms. Respondents are instructed to rate on a 5 point Likert scale from 0 (not at all) to 4 (extremely) how affected they felt by the traumatic event i.e. ICU admission during the preceding five days. There was no modification in the wording, order or content when translating into the language of the respondent. The IES is not a tool for diagnosing PTSD, butinstead detects symptoms indicating a risk of PTSD and has been used in various studies to detect vulnerability to PTSD.[5,6] Sum of the score greater than 26 was considered as severe.[11]

HAD scale is a measure of presence and severity of mild degrees of mood disorder, anxiety and depression. It is a 14-items scale (7 for Anxiety and 7 for depression).[13]

## Results

178 relatives were approached on the fifth day of the ICU stay and 166 consented to participate in the study. The average age of the relatives was 36 yrs (18–67). At the end of two months, 100 of these relatives (60%) answered the telephonic questionnaire.

The characteristics of the relatives and possible stressors considered at admission are shown in Table 1. Out of 166 relatives, 48% had a HAD score >11 on admission suggesting severe anxiety. There was no association of HAD score with gender, patient outcome, working status, age of the patient or mode of payment of the bills. Table 1 shows anxiety during hospitalization to be significantly associated with lower education levels and trauma admission (*P* < 0.05).

**Table 1:** HADS – Anxiety and IES study of 166 relatives

Characteristics	Total no. of relatives	HADS > 11 (%)	*P* value	IES > 26 (%)	*P* value
Sex			0.29		0.28
Male	90	40 (44)		62 (69)	0.22
Female	76	40 (53)		58 (76)	
Total	166	80 (48)		120 (72)	
Working status			0.82		0.16
Working	94	46 (49)		64 (68)	0.15
Non-working	72	34 (47)		56 (78)	
Total	166	80 (48)		120 (72)	
Educational status			<0.05		0.37
Below SSC	29	15 (52)		23 (79)	
Primary level	53	28 (53)		38 (72)	
Secondary level	30	13 (43)		24 (80)	
Graduate and PG	54	24 (44)		35 (65)	
Total	166	80 (48)		120 (72)	
Relationship with the patient			0.24		0.54
Siblings	26	9 (35)		17 (65)	
Children	48	21 (44)		37 (77)	
Spouse	52	29 (56)		39 (75)	
Parents	21	12 (57)		17 (81)	
Others	19	9 (47)		10 (53)	
Total	166	80 (48)		120 (72)	
Diagnosis of patient			<0.05		<0.05
Trauma admissions	49	30 (61)		41 (84)	0.04
Medical admissions	117	50 (43)		79 (68)	
Total	166	80 (48)		120 (72)	
ICU stay			0.88		0.91
<5 days	129	64 (50)		93 (72)	0.39
>5 days	37	16 (43)		27 (73)	
Total	166	80 (48)		120 (72)	
Hospital stay			0.49		0.22
< 11 Days	107	52 (49)		74 (69)	0.19
> 11 Days	59	28 (47)		46 (78)	
Total	166	80 (48)		120 (72)	
IES score			< 0.05		
< 26	46	7 (15)			
> 26	120	73 (61)			
Total	166	80 (48)			
Mode of payment			0.75		<0.05
Self -finance	108	53 (49)		72 (67)	
Financed	58	27 (47)		48 (83)	
Total	166	80 (48)		120 (72)	

IES score > 26 on admission was seen in 120 of the 166 relatives and did not depend on gender, working status, educational level, relationship with patient. The most common group of relatives with IES >26 were the parents. There was a significant (*P* < 0.05) association of IES-R >26 with trauma admission, a HAD score >11 and mode of payment [Table 1]. Relatives of insured or employer payment patients appear more stressed than those who had to settle their bills personally.

Although thelevel of anxiety and degree of stress amongst the relatives was reduced from that seeninitially, one-third relatives (35%) followed-up after two months still showed symptoms like post-traumatic stress reaction consistent with a high risk of PTSD [Table 2]. Persistence of stress symptoms was more in school dropouts, working relatives, parents and those with initial anxiety score >11. On admission a significant association of IES-R >26 was seen with trauma admission, mode of payment; but two months later there was no such connection. The only factor at admission which could statistically predict higher PTSD score on follow-up was a HAD score greater than 11.

**Table 2:** IES study of 100 relatives at the end of second follow-up (after two months)

Characteristics	Total no. of relatives	After five days IES >26 (%)	*P* value	After two months IES >26 (%)	*P* value
Sex			0.85		0.21
Male	57	42 (74)		17 (30)	
Female	43	31 (72)		18 (42)	
Working status			0.92		0.66
Working	60	44 (73)		22 (37)	
Nonworking	40	29 (73)		13 (33)	
Educational status			0.78		0.27
Below SSC	14	11 (79)		8 (57)	
Primary level	35	27 (77)		10 (29)	
Secondary level	17	12 (71)		5 (29)	
Graduate and PG	34	23 (68)		12 (35)	
Relationship with the patient			0.83		0.25
Siblings	19	13 (68)		4 (21)	
Children	34	25 (74)		12 (35)	
Spouse	26	20 (77)		11 (42)	
Parents	11	9 (82)		6 (55)	
Others	10	6 (60)		2 (20)	
Diagnosis of patient			0.003		0.39
Trauma admissions	29	27 (93)		12 (41)	
Medical admissions	71	46 (65)		23 (32)	
ICU stay			0.51		0.71
<5 days	68	51 (75)		23 (34)	
>5 days	32	22 (69)		12 (38)	
Hospital stay			0.60		0.59
<11 days	55	39 (71)		18 (33)	
>11 days	45	34 (76)		17 (38)	
Anxiety score			<0.01		<0.01
<11 days	55	30 (55)		9 (16)	
>11 days	45	43 (96)		26 (58)	
Mode of payment			<0.05		0.46
Self-finance	62	41 (66)		20 (32)	
Financed	38	32 (84)		15 (39)	

The HAD and IES-R scores of the relatives of seven patients who died in the hospital were above the cut off points during admission and at the follow up. The relatives of five patients who died after discharge had both anxiety and IES-R score above the cut off point only at admission and not on the 2-month follow-up. All these patients were significantly older (mean age 75 years) than those who died in the hospital (mean age 45 years).

## Discussion

Our study shows that negative psychological sequelae of an ICU admission in the relatives can persist even at 2 months in more than one third of them and is best predicted by a HAD score greater than 11.

It is intuitive to believe that higher levels of stress and anxiety present in relatives during admission will regress with time. This is corroborated by our finding that the percentage of relatives prone to PTSD related symptoms (suggested by IES-R >26) came down from 78% to 35% within two months. Our study tried to identify these 35% individuals who had persistence of symptoms even at the end of 2 months.

In our earlier study on relatives of trauma patient’s, 14% of them were seen to have persistent PTSD like symptoms at the end of two years.[3] In that study we had not focused on identifying the predisposing factors or relatives with high risk for development of posttraumatic stress reaction. The present study was an attempt to identify predictors for severe psychological stress in both trauma and non-trauma patient’s relatives. Our results showed trauma admission as an important determinant of anxiety and stress disorder only during admission but not on the follow-up indicating other factors during ICU admission or ICU admission itself can lead to persistent psychological distress.

Parents of the patients were more affected and proportionately higher number of them remained stressed even on follow-up. Siblings and distant relatives appeared to recover faster as compared to spouses, parents and children in the follow-up. Persistence of psychological stress in less educated (secondary level and school dropouts) on follow-up could be a reflection of their social support and ability to cope with a major illness in the family. The working status of relatives was not associated with higher IES score during admission but the proportion of working individuals (22 out of 35) on follow-up could indicate an additional burden of balancing working life contributing to the stress.

In our study no relatives participated in end of life decisions, hence the significance of this important variable found in Azoulay’s study could not be commented upon.[6] Twelve patients died in our study, seven in the hospital and five at home. Death of a patient in the hospital resulted in persistently higher scores as against death at home. Though numbers are small, the contribution of end of life discussion with relatives cannot be ruled out and what is conspicuous is the younger age of patients who died in the hospital versus more elderly patients who died at home. Of course the death after discharge could also mean that the relatives had come to terms during this period and accepted the eventuality.

Of the three factors during admission, which statistically contributed to symptoms suggestive of PTSD namely severe anxiety scores, trauma admission and the mode of payment, most unexpected was association of the ability to pay the bills with reduced stress scores, principally because we felt raising finances to pay the bills would be an additional burden. The possible explanation for the surprising association of third party payment with higher IES-R score during admission must be due to the uncertainty or possibility of denials and rejections of payment by insurance companies or employers, because this association was not seen in follow-up when all uncertainties of payment were cleared.

Thus the only predictor on admission for longer-term psychological consequence in our study is the HAD score > 11 and those at higher risk were parents, spouses, children, the less educated and those whose relatives died in the hospital. With studies suggesting family members to perceive reassurance and information as important towards relieving their stress, anticipating and addressing these needs could minimize the stress associated with hospitalization in the ICU.[10] Further research to verify other possible risk factors for PTSD and interventions are vital in preventing development of chronic psychological disorders as stress can hinder family members’ coping and may affect the support and care given to the patient.

There are number of limitations in our study. It was a single center study with a short follow-up of two month. The small number of relatives in this study prevented us from performing multivariate analysis. We also did not have information on prior psychological problems amongst the relatives. This has been shown to be a risk factor for the development of PTSD in ICU patients and may apply to the relatives as it could affect the way these individuals deal with stress.

Both C. Jones and our study have identified the HAD score as a predictor of prolonged negative psychological sequel. Unfortunately various studies have used different cut off points, which may make it difficult to compare data.[5,6] In C Jones *et al*., study an anxiety score 13 was used to indicate severe stress while Pochard F and colleague used 30.[5,6] We used 26 in deference to the original study and to help us compare data with our earlier study on trauma patient’s relatives.

As ICU’s across the world will have different facilities, protocols for care and patient population (different educational levels and financial support) areas of stress will also be different. Identification of common risk predictors will simplify identification of relatives having a higher risk of adverse psychological sequel. The predictive power of early anxiety, particularly a HAD score >11, for later psychologicalmorbidity appears to have universal application with studies from France, UK and now India showing similar results.[5,7]

This could allow interventions focused on these relatives with the aim of preventing the development of delayed posttraumatic stress reaction.

## Conclusion

Having a family member admitted to the ICU is stressful for the whole family. In order to lessen the effect of stress, family members at risk need to be identified. The findings of our study identify relatives with a HAD score >11 to predict long-term psychological distress.
